# Is vagotomy necessary in palliative surgery for incurable advanced gastric cancer?: a retrospective case–control study

**DOI:** 10.1186/s12957-023-03111-9

**Published:** 2023-07-21

**Authors:** Yong-Eun Park

**Affiliations:** Department of Surgery, Yeungnam University Medical Center, 170 Hyeonchungno, Nam-Gu, Daegu, 42415 Korea

**Keywords:** Gastric cancer, Vagotomy, Palliative gastrojejunostomy

## Abstract

**Background:**

The interplay between the nervous system and cancer plays an important role in the initiation and progression of gastric cancer. Few studies have presented evidence that the sympathetic nervous system inhibits the occurrence and development of gastric cancer while the parasympathetic nervous system promotes the growth of gastric cancer. To investigate the effect of vagotomy, which is the resection of a parasympathetic nerve innervating the stomach, on the progression of gastric cancer, a retrospective study was conducted comparing the prognosis of simple palliative gastrojejunostomy (PGJ) and palliative gastrojejunostomy with vagotomy (PGJV).

**Methods:**

From January 01, 2000, to December 31, 2021, the medical records of patients who underwent PGJ or PGJV because of gastric outlet obstruction due to incurable advanced gastric cancer at the Yeungnam University Medical Center were retrospectively reviewed. Patients were divided into two groups: locally unresectable gastric cancer (LUGC) or gastric cancer with distant metastasis (GCDM), according to the reason for gastrojejunostomy, and factors affecting overall survival (OS) were analyzed.

**Results:**

There was no significant difference in surgical outcomes and postoperative complications between the patients with PGJV and patients with PGJ. In univariate analysis, vagotomy was not a significant factor for OS in the GCDM group (HR 1.14, CI 0.67–1.94, *p* value 0.642), while vagotomy was a significant factor for OS in the LUGC group (HR 0.38, CI 0.15–0.98, *p* value 0.045). In multivariate analysis, when vagotomy is performed together with PGJ for LUGC, the OS can be significantly extended (HR 0.25, CI 0.09–0.068, *p* value 0.007).

**Conclusions:**

When PGJ for LUGC was performed with vagotomy, additional survival benefits could be achieved with low complication risk. However, to confirm the effect of vagotomy on the growth of gastric cancer, further prospective studies using large sample sizes are essential.

## Background

Vagotomy is the surgical resection of the vagus nerve that innervates the stomach. In the 1940s, it was commonly performed to reduce acid secretion along with antrectomy under the dogma of “no acid, no ulcer” [1]. However, with the development of drugs such as H2 blocker and proton pump inhibitors, together with the discovery of Helicobacter pylori and its effective eradication treatment, vagotomy is not commonly performed for simple peptic ulcers, but is mainly performed when complications such as bleeding, perforation, and obstruction occur [2–4].

Gastric outlet obstruction (GOO) can be caused not only by benign diseases such as peptic ulcers, but also by malignant diseases such as gastric cancer, duodenal cancer, and pancreatic cancer [5, 6]. When GOO occurs due to a malignant disease, surgery is performed if curative surgical resection is possible. However, depending on the patient’s general condition or disease progression, sometimes only endoscopic stenting or palliative bypass surgery may be performed [7, 8].

Gastrojejunostomy is a commonly performed bypass surgery. Unlike GOO which is caused by benign disease, there is no indication to perform vagotomy together with gastrojejunostomy in GOO due to malignant disease [9–11]. In benign GOO, vagotomy is performed to prevent recurrence and complications from peptic ulcers, especially in the anastomotic area [12]. However, for malignant GOO, where there is no history of peptic ulcers, there is no certain or clear benefit from vagotomy because the risk of ulceration and complications at the anastomosis site after gastrojejunostomy is not as high as gastrojejunostomy for benign GOO.

Interestingly, several studies have reported that vagotomy suppresses gastric tumorigenesis [13–15]. When a normal cell becomes a cancer cell, changes occur in the metabolic processes to support the high demand for energy and biosynthetic precursors, known as metabolic reprogramming. One of the key changes in metabolic reprogramming in cancer cells is increased glucose utilization through glycolysis rather than oxidative phosphorylation, even in the presence of oxygen [16, 17]. In gastric cancer cells, metabolic reprogramming occurs specifically to obtain energy through glutaminolysis, which uses amino acids rather than glycolysis utilizing glucose [18, 19]. One study reported that when part of the stomach was denervated with unilateral vagotomy or botulinum A toxin, the metabolic profile of this area was reversed from glutaminolysis to oxidative phosphorylation and glycolysis [13].

To investigate whether vagotomy can suppress the progression of gastric cancer, a retrospective study was conducted comparing the prognosis of palliative gastrojejunostomy with vagotomy (PGJV) and simple palliative gastrojejunostomy (PGJ) in patients with GOO due to incurable advanced gastric cancer.

## Methods

### Patients and study design

**Fig. 1:**
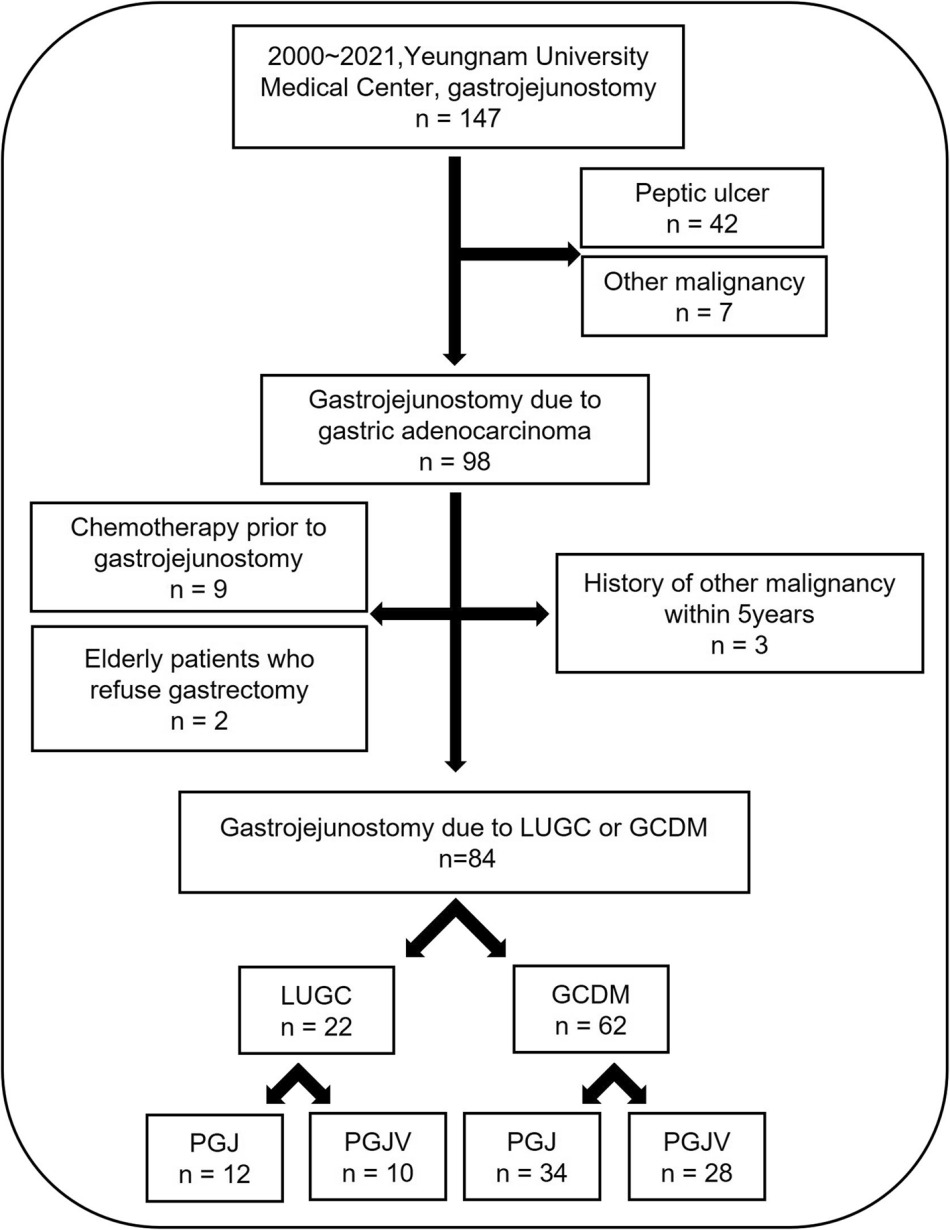
Flow sheet of patient exclusion. From January 01, 2000, to December 31, 2021, the medical records of 147 patients who underwent gastrojejunostomy at the Yeungnam University Medical Center were reviewed. Those who met the exclusion criteria were removed and a final total of 84 patients with gastrojejunostomy due to LUGC or GCDM remained. Abbreviations: *LUGC*, locally unresectable gastric cancer; *GCDM*, gastric cancer with distant metastasis; *PGJ*, simple palliative gastrojejunostomy; *PGJV*, palliative gastrojejunostomy with vagotomy

From January 01, 2000, to December 31, 2021, the medical records of 147 patients who underwent gastrojejunostomy at the Yeungnam University Medical Center were reviewed retrospectively. Patients with partial or complete GOO due to locally unresectable gastric adenocarcinoma or gastric adenocarcinoma with distant metastasis were eligible for this study. Patients who fulfilled the following criteria were excluded: (1) GOO due to peptic ulcer or other malignancy, (2) history of other malignancy within 5 years before gastrojejunostomy, (3) chemotherapy prior to gastrojejunostomy, and (4) resectable gastric cancer without distant metastasis (elderly patients who refuse gastrectomy). According to the eligibility criteria, data belonging to 84 patients were analyzed retrospectively after excluding 63 patients who failed to meet these criteria (Fig. 1).

### Surgical methods for gastrojejunostomy and vagotomy

#### Vagotomy

Both the anterior and posterior vagus nerves were resected at the esophago-gastric junction level. Part of the resected vagus nerve was sent to the pathology department to confirm whether the vagotomy was successful.

#### Gastrojejunostomy

The jejunum about 20 cm away from the Treitz ligament was connected to the dependent portion (distal body, posterior wall without cancer infiltration) of the stomach using the ante-colic method. The afferent loop was directed to the left side of the patient’s abdomen, and the efferent loop was directed to the right side of the patient’s abdomen. One or two tagging sutures were placed between the efferent loop and the stomach wall to prevent kinking. In the case of the open approach, manual anastomosis was performed with vicryl 3–0 and reinforcement suturing was performed with black silk 3–0. In the case of the laparoscopic approach, a small hole was made in the stomach posterior wall and the anti-mesenteric border of the jejunum, and anastomosis was performed using a linear stapler. The common hole was sutured using vicryl 3–0.

### Postoperative care after gastrojejunostomy with or without vagotomy

There was no difference in postoperative management between the PGJ group and the PGJV group. In order to check bowel motility, abdominal X-rays were taken every day from the second day of surgery, and water intake was allowed when gas had passed into the colon. Afterwards, if there was no deterioration of bowel motility on the abdominal X-ray when compared to the previous day, and no discomfort or other patient complications, it was possible to proceed with a diet in the order of a liquid diet to a soft diet. If no specific symptoms or complications occurred, a soft diet was maintained until discharge. One week after discharge, the patient’s dietary discomfort was checked at an outpatient clinic and the decision was made whether to proceed with a regular diet.

### Palliative chemotherapy after bypass surgery

After gastrojejunostomy, if the patient did not want chemotherapy because of their advanced age and poor general condition, only follow-up and symptom control were performed without palliative chemotherapy. If the patient agreed to chemotherapy, palliative chemotherapy was performed. If disease progression was found in the follow-up examination, the chemotherapy regimen was stopped and changed to another regimen. After changing the regimen, if there was a response or stable disease in the follow-up examination, chemotherapy was performed without an additional regimen change. If the disease had progressed in a subsequent follow-up examination, the regimen was changed again. When severe side effects occurred, the regimen was changed after management. If the patient’s general condition deteriorated significantly during chemotherapy, chemotherapy was discontinued in consultation with the patient.

### Variables

Overall survival (OS) was defined as the time (month) from the palliative gastrojejunostomy, with or without vagotomy, to death. The histologic type was determined according to the WHO classification and categorized into two groups: the adenocarcinoma and poorly cohesive carcinoma groups. The adenocarcinoma group included tubular adenocarcinoma with moderately differentiated, poorly differentiated, and signet ring cell components. The poorly cohesive carcinoma group included signet ring cell carcinoma and poorly cohesive carcinoma. Comorbidities were categorized into two groups: those with fewer than three comorbidities and those with more than three comorbidities. The reason for gastrojejunostomy in gastric cancer patients with GOO was reviewed and classified as the locally unresectable group and distant metastatic group. The distant metastatic group included gastric cancer with peritoneal seeding, distant organ metastasis, or distant lymph node metastasis. A mixed pattern of locally unresectable and distant metastasis was also included in the distant metastatic group. Postoperative complications over Clavien-Dindo classification grade three and surgical outcomes such as time to flatus (days) and hospitalization period (days) were collected and analyzed. Postoperative mortality was defined as postoperative death without discharge after gastrojejunostomy.

### Data analysis and statistics

All statistical analyses were performed using SPSS (version 22.0; IBM Corporation, Armonk, NY, USA), with a significance level set at *p* < 0.05. Continuous variables are expressed as mean ± standard deviation and compared using a two-sample *t* test. Categorical variables were expressed as frequencies (percentages) and analyzed using the chi-square test. Univariate and multivariate analyses were conducted using the Cox proportional hazards model to identify prognostic factors. Variables with a *p* value < 0.05 in the univariate analysis were used in the multivariate analysis. The overall survival curve was constructed using the Kaplan–Meier method, while comparison according to vagotomy was performed via the log-rank test.

## Results

A total of 84 patients underwent gastrojejunostomy for GOO due to locally unresectable gastric cancer (LUGC) or gastric cancer with distant metastasis (GCDM). Their average age was 64.6 years, of which 46 (54.8%) were over 65 years of age. There were 65 males (77.4%) and 19 females (22.6%). Twenty-two patients (26.2%) underwent gastrojejunostomy with GOO for LUGC and the remaining 62 patients (73.8%) underwent gastrojejunostomy with GOO for GCDM. After gastrojejunostomy, 58 patients (69%) received palliative chemotherapy at least once, and the remaining 26 patients (31%) refused palliative chemotherapy because of their general condition or advanced age. Following histological classification, 66 patients (78.6%) were placed in the adenocarcinoma group and 18 patients (21.4%) were placed in the poorly cohesive group. Vagotomy was performed in conjunction with gastrojejunostomy in 38 patients (45.2%) but not performed in the remaining 46 patients (54.8%) (Table 1).

**Table 1:** Baseline characteristics of 84 patients

Variables	Description	*n* (%) or mean ± SD
Age (year)	Total	64.6 ± 11.5
	< 65	38 (45.2)
	≥ 65	46 (54.8)
Sex	Female	19 (22.6)
	Male	65 (77.4)
Reason for gastrojejunostomy	LUGC	22 (26.2)
	GCDM	62 (73.8)
Palliative chemotherapy	Not done	26 (31)
	Done	58 (69)
Histologic type	Adenocarcinoma	66 (78.6)
	Poorly cohesive carcinoma	18 (21.4)
Albumin	< 3.5 g/dL	52 (61.9)
	≥ 3.5 g/dL	32 (38.1)
Vagotomy	Not done	46 (54.8)
	Done	38 (45.2)
Comorbidity	< 3	73 (86.9)
	≥ 3	11 (13.1)
Approach method	Open	45 (53.6)
	Laparoscopy	39 (46.4)
Postoperative complication	Negative	73 (88.1)
	Positive	10 (11.9)

*Abbreviations: LUGC* Locally unresectable gastric cancer, *GCDM* Gastric cancer with distant metastasis

The study examined whether there were any differences in the clinicopathological features of the patients according to the reason for gastrojejunostomy. There were no statistically significant differences in patient age, sex, chemotherapy status, histologic type, comorbidities, and the approach method between the LUGC group and the GCDM group (all *p*-values were above 0.05). Similarly, there were no significant differences in clinicopathologic characteristics between the PGJ and PGJV groups, and there was no significant correlation between the reason for gastrojejunostomy and whether vagotomy was performed (Table 2).

**Table 2:** Clinicopathologic features according to whether vagotomy was performed or the reason for palliative surgery

Variables	Description	PGJ (*n* = 46)	PGJV (*n* = 38)	*P* value	LUGC (*n* = 22)	GCDM (*n* = 62)	*P* value
Age	< 65	19 (41.3)	19 (50.0)	0.425	8 (36.4)	30 (48.4)	0.330
	≥ 65	27 (58.7)	19 (50.0)		14 (63.6)	32 (51.6)	
Sex	Female	35 (76.1)	30 (78.9)	0.755	17 (77.3)	48 (77.4)	0.989
	Male	11 (23.9)	8 (21.1)		5 (22.7)	14 (22.6)	
Palliative chemotherapy	Not done	16 (34.8)	10 (26.3)	0.403	9 (40.9)	17 (27.4)	0.240
	Done	30 (65.2)	28 (73.7)		13 (59.1)	45 (72.6)	
Histologic type	Adenocarcinoma	38 (82.6)	28 (73.7)	0.321	17 (77.3)	49 (79.0)	0.863
	Poorly cohesive	8 (17.4)	10 (26.3)		5 (22.7)	13 (21.0)	
Albumin	< 3.5 g/dL	31 (67.4)	21 (55.3)	0.255	13 (59.1)	39 (62.9)	0.752
	≥ 3.5 g/dL	15 (32.6)	17 (44.7)		9 (40.9)	23 (37.1)	
Comorbidity	< 3	40 (87.0)	33 (86.8)	0.988	19 (86.4)	54 (87.1)	0.930
	≥ 3	6 (13.0)	5 (13.2)		3 (13.6)	8 (12.9)	
Approach method	Open	25 (54.3)	20 (52.6)	0.875	13 (59.1)	32 (51.6)	0.546
	Laparoscopy	21 (45.7)	18 (47.4)		9 (40.9)	30 (48.4)	
Vagotomy	None				12 (54.5)	34 (54.8)	0.981
	Positive				10 (45.5)	28 (45.2)	
Reason for gastrojejunostomy	LUGC	34 (73.9)	28 (73.7)	0.981			
	GCDM	12 (26.1)	10 (26.3)				

*Abbreviations: PGJ* Simple palliative gastrojejunostomy, *PGJV* Palliative gastrojejunostomy with vagotomy, *LUGC* Locally unresectable gastric cancer, *GCDM* Gastric cancer with distant metastasis

All values are presented as frequency (percentage)

*p* values were obtained using the chi-square test

There was no significant difference in surgical outcomes and postoperative complications between the PGJV and PGJ groups. In the PGJV group, delayed gastric emptying occurred in one patient, and the hospitalization period for this patient was 25 days, which was 15 days longer than the average. There were three cases of postoperative mortality: one patient in the PGJV group due to infection and worsening renal failure, and one patient in the PGJ group due to pneumonia aggravation, and one patient in the PGJ group due to renal failure and general deterioration. In the PGJV group, one patient had pneumonia, anastomosis site leakage, and infection after surgery, but recovered well and was discharged without mortality (Table 3).

**Table 3:** Postoperative outcomes of palliative gastrojejunostomy with or without vagotomy

Postoperative outcomes	PGJ (*n* = 46)	PGJV (*n* = 38)	*P* value
Overall number of complications	6	7	
Pneumonia	2	2	0.845
Bleeding	2	0	0.193
Leakage	0	1	0.268
Infection	0	2	0.115
Delayed gastric emptying	0	1	0.268
Cardiac arrest	1	0	0.361
Renal failure	1	1	0.891
Time to flatus (days)	3.2 ± 0.9	3.1 ± 1.0	0.924
Hospitalization period (days)	10.0 ± 8.2	10.2 ± 13.1	0.920
Postoperative mortality	2	1	0.673

*Abbreviations: PGJ* Simple palliative gastrojejunostomy, *PGJV* Palliative gastrojejunostomy with vagotomy

All values are presented as mean ± standard deviation or frequency

*p* values were obtained using a two-sample *t* test or the chi-square test

**Fig. 2:**
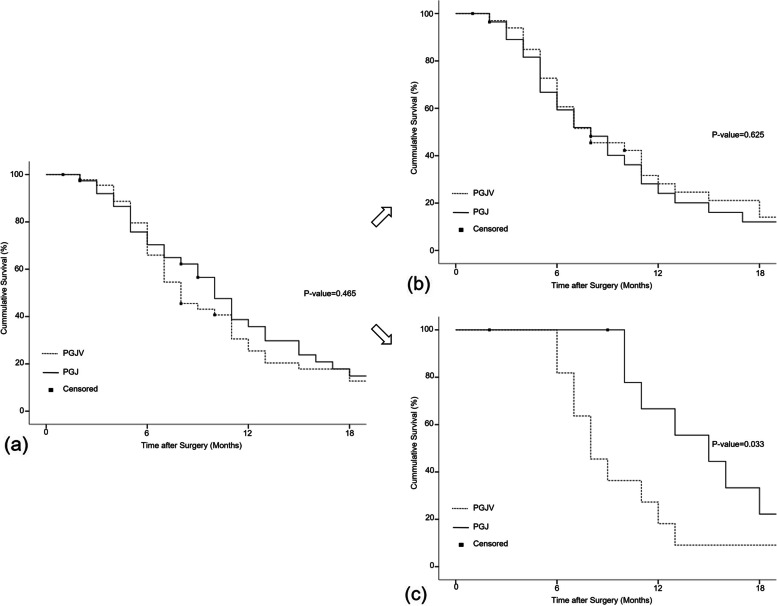
Kaplan–Meier survival curve by vagotomy. There was no significant difference in overall survival according to vagotomy in **a** all patients with GOO (*p* value = 0.465) and **b** in patients with GOO due to GCDM (*p* value = 0.625). Vagotomy was a significant protective factor for overall survival **c** in patients with GOO due to LUGC (*p* value = 0.033). Abbreviations: *GOO*, gastric outlet obstruction; *GCDM*, gastric cancer with distant metastasis; *LUGC*, locally unresectable gastric cancer; *PGJ*, simple palliative gastrojejunostomy; *PGJV*, palliative gastrojejunostomy with vagotomy

Cox proportional-hazard analysis was performed to investigate factors affecting OS in patients who underwent PGJ due to GOO by incurable advanced gastric cancer. Univariate analysis revealed that age, chemotherapy, and comorbidity were statistically significant factors for OS. Vagotomy was not a significant factor in all patients. In multivariate analysis, chemotherapy was the only factor significantly affecting OS. After dividing the groups according to the reason for PGJ, the factors affecting the OS in each group were investigated. In the GCDM group, chemotherapy was the only significant factor, whilst age, comorbidity, and vagotomy had no significant effect on OS. In the LUGC group, as a result of univariate analysis, chemotherapy, and vagotomy were significant factors affecting OS. Surprisingly, in multivariate analysis, both chemotherapy and vagotomy were protective factors for OS in the LUGC group. This suggests that when vagotomy is performed together with PGJ for LUGC, the OS can be significantly extended (HR 0.25, CI 0.09–0.68, *p* value 0.007) (Table 4). Figure 2 shows the survival curves by vagotomy in each group (Fig. 2).

**Table 4:** Univariate and multivariate analysis of overall survival in gastric cancer patients with outlet obstruction

Variables	Total (*n* = 84)				LUGC (*n* = 22)				GCDM (*n* = 62)	
	Univariate		Multivariate		Univariate		Multivariate		Univariate	
	HR (95% CI)	*P* value	HR (95% CI)	*P* value	HR (95% CI)	*P* value	HR (95% CI)	*P* value	HR (95% CI)	*P* value
Age ≥ 65	1.63 (1.02–2.60)	0.040	1.42 (0.88–2.29)	0.156	2.83 (0.97–8.29)	0.058			1.49 (0.87–2.55)	0.148
Male	0.95 (0.55–1.63)	0.846			0.83 (0.27–2.54)	0.738			0.95 (0.51–1.77)	0.859
Chemotherapy	0.27 (0.15–0.48)	0.000	0.31 (0.16–0.57)	0.000	0.18 (0.05–0.66)	0.009	0.10 (0.03–0.43)	0.002	0.21 (0.10–0.44)	0.000
Poorly cohesive (ref. adenocarcinoma)	1.71 (0.99–2.97)	0.056			1.61 (0.56–4.67)	0.378			1.84 (0.96–3.52)	0.067
Albumin ≥ 3.5 g/dL	1.22 (0.76–1.97)	0.411			1.25 (0.49–3.16)	0.654			1.20(0.69–2.11)	0.514
Vagotomy	0.85 (0.54–1.34)	0.487			0.38 (0.15–0.98)	0.045	0.25 (0.09–0.68)	0.007	1.14 (0.67–1.94)	0.642
Comorbidity ≥ 3	2.21 (1.10–4.41)	0.025	1.32 (0.64–2.74)		3.76 (0.98–14.38)	0.369			1.96 (0.86–4.43)	0.567
Laparoscopy	1.07 (0.67–1.72)	0.775			1.25 (0.46–3.39)	0.664			0.98 (0.57–1.69)	0.952
Postoperative complication	1.99 (0.95–4.19)	0.069			3.80 (0.78–18.45)	0.097			1.81 (0.77–4.26)	0.177
GCDM (ref. LUGC)	0.69 (0.41–1.17)	0.166								

*Abbreviations: LUGC* Locally unresectable gastric cancer, *GCDM* Gastric cancer with distant metastasis

All values are presented as the hazard ratio (HR) and 95% confidence interval for HR

*p* values were obtained by the cox-proportional hazard model

## Discussion

The interplay between the nervous system and cancer plays an important role in the initiation and progression of cancer. The sympathetic nervous system inhibits the occurrence of gastric cancer, and the distribution of sympathetic nerves in gastric cancer tissue was significantly reduced compared to normal gastric tissue [20]. In addition, the density of sympathetic nerve fibers in advanced gastric cancer patients with pT4 was significantly lower than that of pT1-3 [21]. The parasympathetic nervous system secretes acetylcholine, which acts on muscarinic acetylcholine receptor 3 to promote the growth of gastric cancer [22]. Therefore, both the sympathetic and parasympathetic nerves of the autonomic nervous system affect the occurrence and growth of gastric cancer through antagonism [23, 24].

**Fig. 3:**
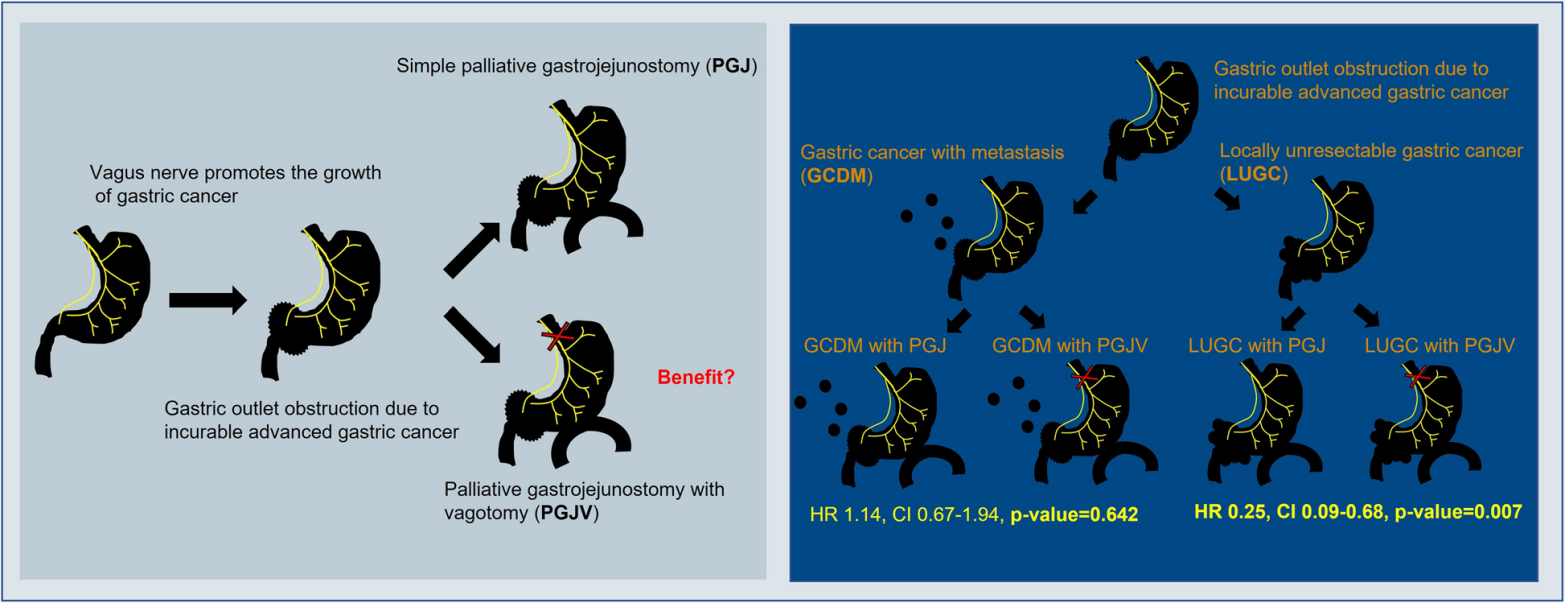
Overview of background and results. To investigate the effect of vagotomy on the progression of gastric cancer, a retrospective study was conducted comparing the prognosis of PGJ and PGJV. The study results showed that when vagotomy is performed together with PGJ for locally unresectable gastric cancer, the overall survival can be significantly extended. Abbreviations: *GCDM*, gastric cancer with distant metastasis; *LUGC*, locally unresectable gastric cancer; *PGJ*, simple palliative gastrojejunostomy; *PGJV*, palliative gastrojejunostomy with vagotomy

Based on these studies on the interaction between gastric cancer and the autonomic nervous system, it can be concluded that the vagus nerve, a parasympathetic nerve involved in gastric motion, promotes the progression of gastric cancer. Therefore, the author hypothesized that blocking the vagus nerve would inhibit the progression of gastric cancer and designed this study. Surprisingly, the study found that the OS of patients with PGJV due to LUGC was significantly higher than that of patients with PGJ alone due to LUGC, which suggests that vagotomy inhibits the growth of gastric cancer (Table 4) (Fig. 3).

To further explain, vagotomy should be performed without gastrectomy in order to examine the exact effect of a parasympathetic blockade on the growth of gastric cancer. Therefore, in this study, patients who underwent PGJ or PGJV for incurable advanced gastric cancer were targeted to prove the exact effect of vagotomy.

PGJ is a commonly used palliative bypass surgery for GOO due to incurable advanced gastric cancer. Gastric cancer guidelines describe PGJ as follows. The NCCN guideline: one of several options to alleviate symptoms or bypass when obstruction occurs in unresectable locally advanced gastric cancer or recurrent or metastatic gastric cancer [9]. The Korean guideline: PGJ can be performed in GOO caused by unresectable gastric cancer. However, PGJ should be determined after a multidisciplinary assessment of the patient’s performance status, projected clinical course, and preference [11]. The Japanese guideline: As a non-curative surgery to relieve obstructive symptoms, palliative gastrectomy or gastrojejunostomy is selected depending on the resectability of the primary tumor and/or surgical risk [10]. Unfortunately, in all the above guidelines, there was no criterion for performing a vagotomy with gastrojejunostomy. If the effect of a parasympathetic blockade on the growth of gastric cancer is proven through further large-sampled prospective studies, vagotomy should be recommended in the palliative surgery guidelines.

Previous studies of PGJ for incurable advanced gastric cancer reported postoperative chemotherapy, performance status of the patient, and severity of peritoneal seeding as factors affecting OS [25, 26]. These factors affect the OS but are uncorrectable factors except for postoperative chemotherapy. In this study, both chemotherapy and vagotomy had a statistically significant effect on OS, and vagotomy is a correctable factor that can be easily performed with PGJ at low risk (Tables 2 and 4). However, vagotomy had a significant effect on OS only in the LUGC group and had no significant effect on OS in the GCDM group (Table 4). A possible reason for these results is that gastric denervation by vagotomy, and the resulting inhibitory effect on gastric cancer progression, does not apply to cases that have already metastasized to the peritoneum or other organs. Therefore, vagotomy with PGJ should be selectively applied in LUGC cases without distant metastasis.

In this study, prognostic factors were analyzed not only in the overall patients with incurable advanced gastric cancer but also in two groups based on the reason for gastrojejunostomy, identifying prognostic factors that impact OS in each group (Table 4). In addition, as previously mentioned, cases where gastrectomy was performed were excluded in this study to specifically examine the effects of vagotomy on gastric cancer. In contrast, previous studies of PGJ for incurable advanced gastric cancer considered the reason for gastrojejunostomy as one of the prognostic factors but have not divided the patients according to it and not analyzed the prognostic factor in each group [25, 26]. Furthermore, patients who underwent palliative gastrectomy as well as PGJ were included [25]. Therefore, in future studies investigating the impact of vagotomy on gastric cancer, it will be necessary to consider the grouping according to the degree of gastric cancer progression and the implementation of gastrectomy.

Currently, treatment for incurable advanced gastric cancer consists mainly of chemotherapy and radiotherapy, and the role of surgery in palliative treatment is limited [9–11, 27]. This is because palliative treatment focuses not only on survival gain, but also on the patient’s symptom resolution and quality of life, while surgical treatment requires general anesthesia and can cause additional pain and postoperative morbidity and mortality [28]. However, considering that gastric cancer is the fifth most common cancer worldwide and most gastric cancers are already advanced at the time of diagnosis, it is not uncommon in clinical practice for incurable advanced gastric cancer to require surgery [29, 30]. In this study, there was no difference in OS according to the reason for gastrojejunostomy. However, when an additional surgical procedure called vagotomy was performed, the results showed that vagotomy was a protective factor for the OS in LUGC. In situations where surgery is unavoidable during palliative care, research on surgical methods that can obtain additional benefits, such as stomach partitioning gastrojejunostomy or vagotomy, is necessary [31–33].

This study concluded that when performing PGJ in locally unresectable gastric cancer, additional vagotomy yielded a survival benefit. However, to date, there have been few studies analyzing the impact of vagotomy on gastric cancer patients in clinical practice, apart from animal experiments [13–15]. This is likely because the effects of para-sympathetic nerve blockade through vagotomy in gastric cancer have not been extensively studied, and there is a lack of relevant guidelines. Considering the research on the interaction between the nervous system and gastric cancer, it will be necessary to conduct further studies not only on surgical interventions like vagotomy but also on endoscopic methods such as botulinum toxin injections and pharmacological approaches that modulate the stimulation from the nervous system.

Neo-adjuvant chemotherapy before gastrojejunostomy was excluded from this study. This is because chemotherapy prior to gastrojejunostomy means that GOO occurred due to disease progression, even though chemotherapy was performed after a diagnosis of advanced gastric cancer. Thus, cases where chemotherapy was performed prior to gastrojejunostomy were omitted, to exclude deterioration of the patient’s condition due to previous chemotherapy and to equalize the initial diagnosis time.

This study had several limitations. First, it was a retrospective and small sample-sized single-center analysis; therefore, it may have been biased. Second, there may be differences in the effectiveness of chemotherapy according to the change of regimen and anticancer drugs between the past and the present. On the other hand, there is no significant difference between the past and the present in terms of surgical methods, indications, and vagotomy methods. Third, in the palliative management of cancer patients, the quality of life is also an important factor, but this was not taken into account during this study. Therefore, in order to investigate the clinical advantages of vagotomy in palliative surgery, future studies on vagotomy should also evaluate the effect of vagotomy on the quality of life.

## Conclusions

Surgery is inevitable during the palliative treatment of incurable advanced gastric cancer, and research regarding surgical methods to obtain additional benefits is also necessary. In this study, when PGJ for LUGC was performed with vagotomy, additional survival benefits could be achieved with low complication risk, which suggests that vagotomy inhibits the growth of gastric cancer.

## Data Availability

The anonymized data used and/or analyzed during the current study are available from the corresponding author upon reasonable request.

## Abbreviations

GOO: Gastric outlet obstruction
PGJ: Simple palliative gastrojejunostomy
PGJV: Palliative gastrojejunostomy with vagotomy
LUGC: Locally unresectable gastric cancer
GCDM: Gastric cancer with distant metastasis
